# Cooked Bean (*Phaseolus vulgaris* L.) Consumption Alters Bile Acid Metabolism in a Mouse Model of Diet-Induced Metabolic Dysfunction: Proof-of-Concept Investigation

**DOI:** 10.3390/nu17111827

**Published:** 2025-05-28

**Authors:** Tymofiy Lutsiv, Vanessa K. Fitzgerald, Elizabeth S. Neil, John N. McGinley, Hisham Hussan, Henry J. Thompson

**Affiliations:** 1 Cancer Prevention Laboratory, Colorado State University, Fort Collins, CO 80523, USA; tymofiy.lutsiv@colostate.edu (T.L.); vanessa.fitzgerald@colostate.edu (V.K.F.); elizabeth.neil@colostate.edu (E.S.N.); john.mcginley@colostate.edu (J.N.M.); 2 Graduate Program in Cell and Molecular Biology, Colorado State University, Fort Collins, CO 80523, USA; 3 Department of Internal Medicine, University of California, Davis, Sacramento, CA 95817, USA; hhussan@ucdavis.edu

**Keywords:** bile acid, gut microbiome, bile acid sequestrant, pulses, bean, MASLD, obesity

## Abstract

**Background/Objectives**: Metabolic dysregulation underlies a myriad of chronic diseases, including metabolic dysfunction-associated steatotic liver disease (MASLD) and obesity, and bile acids emerge as an important mediator in their etiology. Weight control by improving diet quality is the standard of care in prevention and control of these metabolic diseases. Inclusion of pulses, such as common bean, is an affordable yet neglected approach to improving diet quality and metabolic outcomes. Thus, this study evaluated the possibility that common bean alters bile acid metabolism in a health-beneficial manner. **Methods**: Using biospecimens from several similarly designed studies, cecal content, feces, liver tissue, and plasma samples from C57BL/6 mice fed an obesogenic diet lacking (control) or containing cooked common bean were subjected to total bile acid analysis and untargeted metabolomics. RNA-seq, qPCR, and Western blot assays of liver tissue complemented the bile acid analyses. Microbial composition and predicted function in the cecal contents were evaluated using 16S rRNA gene amplicon and shotgun metagenomic sequencing. **Results**: Bean-fed mice had increased cecal bile acid content and excreted more bile acids per gram of feces. Consistent with these effects, increased synthesis of bile acids in the liver was observed. Microbial composition and capacity to metabolize bile acids were markedly altered by bean, with greater prominence of secondary bile acid metabolites in bean-fed mice, i.e., microbial metabolites of chenodeoxycholate/lithocholate increased while metabolites of hyocholate were reduced. **Conclusions**: In rendering mice resistant to obesogenic diet-induced MASLD and obesity, cooked bean consumption sequesters bile acids, increasing their hepatic synthesis and enhancing their diversity through microbial metabolism. Bean-induced changes in bile acid metabolism have potential to improve dyslipidemia.

## 1. Introduction

In the age of rapidly rising metabolic dysfunction-associated co-morbidities, metabolic dysfunction-associated steatotic liver disease (MASLD) is of particular concern. Currently, over 30% of adults are affected by MASLD, with a significant economic burden worldwide, exceeding USD 100 billion in the US alone [1]. The term “MASLD” has recently replaced the literature-rich non-alcoholic fatty liver disease (NAFLD) [2,3,4]. MASLD is associated with a wide range of diseases involving dysregulated metabolism, including more severe pathologies of the liver (metabolic dysfunction-associated steatohepatitis (MASH), cirrhosis, hepatocellular carcinoma), albeit the leading cause of mortality in patients with MASLD remains cardiovascular disease (CVD) accompanied by dyslipidemia [5]. Lifestyle interventions, such as regular intake of high-quality diets (adherent to the Dietary Guidelines [6]) and increased physical activity to achieve weight loss and maintenance, remain central to the management of MASLD and point to the predominant culprit in its incidence—excess caloric intake. Therefore, switching from Westernized dietary patterns to the consumption of plant-based protein and increased dietary fiber and whole foods rather than ultra-processed foods is the first line of recommendation for preventing and controlling MASLD [7,8].

Consumption of common bean, the most consumed representative of grain legumes, i.e., pulses, has been reported to have beneficial effects on multiple chronic diseases [9,10]. Though acknowledged as a concentrated source of dietary protein, bean also provides equivalent amounts of dietary fiber [11]. While dietary fiber is not considered a nutrient, it is well established that its presence in the diet has marked effects on health, especially via effects on intestinal microbiota [12,13]. The chronic diseases impacted, in addition to MASLD, include obesity, type 2 diabetes, CVDs, and several types of cancer; however, the mechanism(s) underlying these effects has not been fully elucidated [14]. While a number of pathways have been implicated, e.g., hepatic de novo lipogenesis [15], ceramide-mediated signaling [16], and mTOR network signaling [17], limited effort has been directed to linking such effects to bile acids.

The role of bile acids in host metabolism extends far beyond lipid digestion and absorption, to physiologic and pathophysiologic effects encompassing inflammatory responses, effects on vasculature, glucose metabolism, insulin sensitivity, energy expenditure, and other mediators of metabolic health [18,19]. Bile acids are produced from cholesterol and conjugated with amino acids (e.g., taurine in mice) to form bile salts in the liver. The bile acids synthesized in the liver are deemed primary and comprise cholic and chenodeoxycholic acids in humans [20]. Murine livers additionally synthesize hyocholic, ursodeoxycholic, and α-/β-muricholic acids [20,21,22]. Those bile acids are then secreted into the gallbladder and released into the small intestine within bile, aiding in dietary lipid emulsification/absorption. Most bile acids are reabsorbed via enterohepatic circulation in the lower ileum, while the remaining bile acids interact with intestinal microorganisms in the colon. Microbiota deconjugate and re-conjugate bile acids, conduct their dehydroxylation/hydroxylation, dehydrogenation, oxidation, epimerization, and other modifications [22], producing secondary bile acids, such as deoxycholic, lithocholic, hyodeoxycholic, ω-muricholic acids, etc. Further biotransformation of secondary bile acids also takes place, seldom distinguished in the scientific literature as tertiary bile acids [23,24,25]. Enterohepatic circulation continues along the large intestine, and unabsorbed bile acids/salts are excreted with feces. Some primary and secondary bile acids also reach the systemic circulation, extending their function as signaling molecules beyond the gastrointestinal tract and liver. Patients with metabolic diseases, like MASLD and obesity, often exhibit hypercholesterolemia and elevated bile acid levels (especially conjugated primary species), so among therapeutic approaches, complementing dietary modifications and exercise, prescription of bile acid sequestrants is common [19,26]. Drugs such as cholestyramine bind bile acids in the gastrointestinal tract and eliminate them with feces, resulting in greater redirection of hepatic cholesterol to bile acid synthesis to compensate for bile acid loss. First- and second-generation bile acid sequestrants are based on plant-derived polysaccharides. Importantly, polysaccharide and protein biopolymers in common bean have been reported to act as bile acid sequestrants [27].

The functional repertoire of bile acids is enhanced by the gut microbiota, which show bidirectional effects as bile acids affect intestinal bacterial communities and the latter metabolize and alter bile acid composition and concentration in the gut [25,28]. Common bean is an abundant source of total dietary fiber as assessed by the integrated method of analysis [29]. In fact, to our knowledge, common bean has one of the highest concentrations of total dietary fiber per 100 kcal edible portion of any category of whole food [30]. Not only does this indicate that bean consumption has strong potential to exert effects on the composition of the gut microbiome, as has been reported in humans and mice, but it is likely the lower digestibility of bean protein in conjunction with its dietary fiber content will exert unique effects on the composition and function of the colon microbiome [31].

The work reported herein is a proof-of-concept investigation. Conceptualization was prompted by several clinical/feeding studies’ observations in which the consumption of common bean renders an organism resistant to severe complications from obesogenic lifestyles, particularly related to dyslipidemia [15,16,17,32]. Biospecimens from previous studies were interrogated to discern whether sufficient evidence exists to support hypothesis testing in prospective preclinical and clinical investigations.

## 2. Materials and Methods

### 2.1. Experimental Design

Experiments were conducted in male and female C57BL6/J mice (cat. 000664) from the Jackson Laboratory (Barr Harbor, ME, USA) at 20-day-old age in multiple studies. Mice had ad libitum access to food and filtered water in solid-bottom polycarbonate mouse cages with a 12 h light/dark cycle at 27.5 ± 2 °C ambient temperature. Prior to eight weeks of age, animals were adapted to husbandry conditions and diet, which was a purified powdered diet providing 32.5% kcal from fat. At 8 weeks of age, mice were assigned to experimental diet groups using staggered randomization by body weight and fed their respective diet for 12–14 weeks.

Experimental diets were isocaloric and formulated to maintain equal macronutrient proportions, weight by weight, according to the AIN-93G recommendations [33,34]. The bean seed was cooked, processed with leachate, puréed, freeze-dried, and milled into a homogenous fine powder. In contrast, control contained only purified ingredients. For more information on the formulations of experimental diets, please refer to Supplementary Table S1.

At necropsy, mice were anesthetized via isoflurane inhalation to a surgical plane of anesthesia, blood was collected via cardiac puncture using a 25 or 26 g needle and 1 cc syringe, and transferred into an EDTA-coated microtainer tube. The animals were then euthanized by cervical dislocation. The blood was spun at 2000× *g* for 10 min, the plasma was collected, transferred to a cryovial, and snap-frozen in liquid nitrogen. Liver was excised, placed in a sample bag, and snap-frozen in liquid nitrogen. The cecal contents were gently harvested and thoroughly mixed into a homogenous mixture, placed in a sterile cryovial, and snap-frozen in liquid nitrogen for subsequent DNA extraction. The liver, cecal contents, and plasma samples were stored at −70 °C. All animal work was approved by the CSU IACUC (Colorado State University, Institutional Animal Care and Use Committee protocol number 1431).

### 2.2. Measurement of Total Bile Acids

Cecal contents, feces, liver tissue, and plasma samples were subjected to the colorimetric total bile acid assay using the Mouse Total Bile Acid kit (cat. 80471, Crystal Chem, Inc.; Elk Grove Village, IL, USA). Feces from 24 h collection were placed in 2 mL cryovials and stored at −70 °C. Dry matter was performed on each sample in duplicate. Briefly, each sample was weighed out and dried in a 90 °C oven for 24 h, the samples were removed and cooled for 30 min in a desiccator and weighed. This process was repeated until the samples were at a constant dry weight. From this, the percent dry matter was calculated and the average of the duplicates was used. Tissues were weighed out and 75% ethanol was added in a volume 10 times greater than the amount of material used for cecal and fecal material, and 5–8 times greater volume for ground frozen liver. Samples were sonicated for 2 h at 50 ± 3 °C, after which they were centrifuged at 4000× *g* for 10 min. EDTA-treated plasma samples were used directly. The assay procedure was performed on a 96-well plate with each sample assayed in duplicate according to the manufacturer’s protocol. In addition to the provided calibrator, a control of known bile acid concentration for mice was also used (cat. 80473; Crystal Chem, Inc.; Elk Grove Village, IL, USA). Deionized water was used as a blank. Samples were incubated with Reagent CC1 (nitrotetrazolium blue (NBT) solution) for 5 min at 37 °C, after which absorbance was measured at 540 nm. Then, Reagent CC2, containing 3α-hydroxysteroid dehydrogenase, was added, and the mixture was incubated for 5 min at 37 °C. 3α-hydroxysteroid dehydrogenase converts 3α-OH bile acids into 3-keto steroids in the presence of NAD^+^, forming an equimolar quantity of NADH, which, in turn, reacts with NBT in the presence of diaphorase enzyme, forming a formazan dye. After adding the stop solution, absorbance measurement was conducted at 540 nm to detect the newly formed dye. Plates were run in the microplate reader (SpectraMax M5, Molecular Devices, LLC; San Jose, CA, USA), detecting formazan dye at 540 nm, which is proportional to the concentration of bile acids. Blank-corrected difference in two absorbances is proportional to the bile acid titer of the original sample, and using calibrator/control absorbance, values were extrapolated into µM levels of detected bile acids. The amount of bile acids detected was corrected for the percentage of dry matter in the cecal, fecal, and liver tissue samples, followed by the density correction of the volume of ethanol used. Values were normalized for the total percent dry matter of cecal and liver weights collected during necropsy.

### 2.3. Metabolomic Profiling

Samples of plasma, cecal contents, and liver were delivered to Metabolon Inc. (Morrisville, NC, USA) for metabolomic analysis using ultrahigh-performance liquid chromatography–tandem mass spectroscopy (UPLC-MS/MS). Given our previous findings that females were more responsive to the bean effect [15], this study was originally designed to focus on 7 samples from males with female samples pooled into 1 sample per group for cost-effective purposes, resulting in *n* = 8 per experimental group (control and bean) per tissue. Metabolon used Waters ACQUITY ultra-performance liquid chromatography (UPLC) with a Thermo Scientific Q-Exactive high-resolution/accurate mass spectrometer interfaced with a heated electrospray ionization (HESI-II) source and Orbitrap mass analyzer operated at 35,000 mass resolution [35]. Samples were analyzed on Metabolon’s global untargeted metabolomics platform (HD4) using their standard statistical approaches. Briefly, the resulting peak area-under-the-curve data were median-scaled (each compound across samples is corrected to have a median of 1.00, normalizing each data point proportionately). Then, missing values were imputed by replacing them with the respective compound’s observed minimum value. For plasma samples, the resulting data were additionally normalized for the extracted volume differences and rescaled to have a median of 1.00. To determine the statistical differences between control and bean, Welch’s two-sample *t*-test was performed with additional adjustment to decrease the false discovery rate using the Benjamini–Hochberg procedure. Separately, for compounds identified by Metabolon as bile acids, median-scaled imputed data were subjected to principal coordinates analysis (PCoA) using the Bray–Curtis dissimilarities and Jaccard distances. Visual separation or lack thereof was confirmed using permutational multivariate analysis of variance (PERMANOVA) to compare the variation between groups to the variation within groups (differences in the means/centroids) and a test for multivariate dispersion (PERMDISP) to check whether differences between groups arise from dispersion of the data.

### 2.4. RNA Isolation and RNA-Seq Analysis

Frozen liver tissue was ground to powder under liquid nitrogen to perform RNA extraction with the RNeasy mini-kit following the manufacturer’s protocol (cat. 74104, QIAGEN, Inc., Germantown, MD, USA). Integrity of isolated RNA was determined using the Experion automated electrophoresis station (Bio-Rad Laboratories, Inc., Hercules, CA, USA). Liver RNA samples were then submitted for cDNA library construction and RNA sequencing (Illumina, Inc., San Diego, CA, USA) to the Genomics and Microarray Core at the University of Colorado-Anschutz Medical Campus (Aurora, CO, USA). Raw sequencing data were processed using CLC Genomics Workbench software, version 23 (QIAGEN, Redwood City, CA, USA), and resulting differentially expressed genes (DEGs) datasets were further evaluated with Ingenuity Pathway Analysis (IPA) software, v127006219, for subsequent analysis (QIAGEN, Redwood City, CA, USA). Out of 25,526 DEGs, 21,797 DEGs were protein-coded and used for the analysis. IPA’s Core Analysis was performed on the dataset of DEGs with maximum mean between the groups greater than 10 TPM and *p*-value < 0.05. Multiple DEGs mapping to a single molecule in IPA were resolved by the highest mean TPM value between the groups. Thus, 2804 analysis-ready molecules were used for the IPA’s Core Analysis. Briefly, expression patterns of the DEGs were cross-referenced with the published studies worldwide within the QIAGEN Ingenuity Knowledge Base, and *p*-values of overlap between the observed DEGs patterns and their associations were calculated. Similarly, activation *z*-scores measuring the prediction strength of the DEGs effects (activation or inhibition) were calculated based on the direction of DEGs expression changes. Mapped functional associates were deemed significant with the |*z*|-score ≥ 2 and the *p*-value < 0.05 of the overlap between our experimentally observed dataset and the QIAGEN Ingenuity Knowledge Base.

### 2.5. Quantitative Real-Time PCR Analysis

An amount of 1 µg of total RNA isolated from liver tissue samples (see Section 2.4) was used for cDNA synthesis with Superscript II reverse transcriptase (Invitrogen, Carlsbad, CA, USA). DNA oligo-primers were previously synthesized (Integrated DNA Technologies, Coralville, IA, USA) [36]. Quantitative real-time PCR (qPCR) was performed on an iCycler iQ5 (Bio-Rad, Hercules, CA, USA) using optical-grade 96-well plates (Thermo Fisher Scientific, Waltham, MA, USA). As previously reported [37], each 20 µL PCR reaction was composed of 10 µL 2X SYBR green Supermix (Bio-Rad, Hercules, CA, USA), 1.5 µL of 4 µM forward and reverse primers, 2 µL synthesized cDNA, and 5 µL nuclease-free water. The PCR conditions were as follows: 95 °C for 1.5 min, followed by 40 cycles of 95 °C for 15 s and 56.5 °C for 1 min. Fluorescent products were detected at the last step of each cycle. qPCR was followed by a melt curve analysis to verify primer specificity. Samples were run in triplicate, and the relative expressions of FXR and SHP were calculated by normalizing the threshold cycle (Ct) values to β-actin (*Actb*) gene expression. Expression of β-actin was set up in reactions that included 10 µL 2X TaqMan Fast Advanced Master Mix, 1 µL 20X TaqMan Gene Expression Assay (β-actin: Mm00607939; Thermo Fisher Scientific, Waltham, MA, USA), 2 µL synthesized cDNA, and nuclease-free water to 20 µL total volume. The TaqMan thermocycling conditions were 50 °C for 2 min, 95 °C for 2 min, followed by 40 cycles of 95 °C for 3 s and 60 °C for 30 s. The fold change in expression relative to the control-fed animals was computed by the negative value of the ΔΔCt method as previously described [38].

### 2.6. Western Blot Analysis

Frozen liver tissue samples were ground to a powder under liquid nitrogen, weighed into pre-cooled microcentrifuge tubes, and subjected to protein lysate extraction as previously described [39]. Protein lysate concentration was determined by the bicinchoninic acid assay (BCA) method [40]. Nanocapillary Immuno-electrophoresis was performed using the Jess instrument (ProteinSimple, San Jose, CA, USA) as previously described [41] with the following changes: final concentration of the protein lysates was 0.7 mg/mL when probed with anti-FGF15 and 0.2 mg/mL for all other targets, and each primary antibody was incubated for 120 min. The following primary antibodies were used: mouse monoclonal anti-FXR/NR1H4 (D-3; cat. SC-25309), mouse monoclonal anti-SHP (H-5; cat. SC-271511), mouse monoclonal anti-FGF-15 (D-9; cat. SC-514647), rabbit polyclonal anti-CYP7A1 (E-10; cat. SC-518007), and mouse monoclonal anti-CYP7B1 (WW-H9; cat. SC-134309) from Santa Cruz Biotechnology, Inc. (Dallas, TX, USA). Data were normalized by dividing the target protein peak area-under-the-curve by the corrected total protein area-under-the-curve within each sample capillary using the Compass Software for Simple Western (v6.1.0; ProteinSimple, San Jose, CA, USA).

### 2.7. Microbiome Analysis Using 16S rRNA Gene Amplicon Sequencing

Total DNA was extracted from cecal contents with the QIAamp PowerFecal DNA kit (cat. 51804, Qiagen, Germantown, MD, USA) according to the manufacturer’s protocol, and its purity and concentration were determined by NanoDrop (Thermo Fisher Scientific, Waltham, MA, USA). The V4 region of the 16S rRNA gene was amplified and sequenced to construct paired-end sequencing libraries using the 515F-806R primer set following the Earth Microbiome Project protocols [42], followed by sequencing using the MiSeq Reagent Kit v2 2 × 250 bp on an Illumina MiSeq instrument (Illumina, Inc., San Diego, CA, USA) housed at the Next-Generation Sequencing Facility at Colorado State University.

Bioinformatic processing of the obtained forward and reverse paired-end sequence reads was performed with QIIME 2 platform, version 2024.10 [43]. Raw sequence data were demultiplexed and quality-filtered via q2-demux plugin. Denoising was performed with the DADA2 pipeline (q2-dada2) [44]. Taxonomy was assigned to the resulting amplicon sequence variants (ASVs) using a Naive Bayes classifier (via q2-feature-classifier plugin [45]) pre-trained on Greengenes2 (16S rRNA, version 2022.10) marker gene reference database trimmed to the V4 domain (bound by the 515F/806R primer pair) with 99% sequence identity threshold [46,47]. The dataset was filtered to remove all features annotated as “mitochondria” and “chloroplast”. A rooted phylogenetic tree was built using FastTree [48] and MAFFT [49] alignment via q2-phylogeny plugin. ASVs feature table was collapsed to the Genus taxonomy level and subjected to the differential abundance analysis using ANCOM-BC method [50] in QIIME 2.

Functional attributes of the identified microbial communities were predicted using Phylogenetic Investigation of Communities by Reconstruction of Unobserved States 2 (PICRUSt2) pipeline, version 2.4.1 [51]. With the ASV dataset as an input, PICRUSt2 performs phylogenetic placement by aligning ASVs to the reference 16S sequences (HMMER, www.hmmer.org) and incorporating them into the reference tree (evolutionary placement algorithm (EPA)-NG and genesis applications for phylogenetic placement analysis (GAPPA) [52,53], followed by the hidden-state prediction of gene families (castor R package [54]) and, finally, generation of metagenomic predictions and tabulation of pathways’ inferences and abundances (Minimal set of Pathways (MinPath) [55] and MetaCyc [56]. Differential abundance analysis of microbiome data was performed in QIIME 2 using ANCOM-BC method [50].

### 2.8. Microbiome Analysis Using Shotgun Metagenomic Sequencing

Whole-genome DNA sequencing was performed on total DNA extracted from cecal samples pooled from 20 animals total (*n* = 3 per experimental group). Libraries were prepared and sequenced on an Illumina NovaSeq 6000 system at the Genomics Shared Resource at the University of Colorado, Anschutz Medical Campus (Aurora, CO, USA). Specifically, 100 ng of genomic DNA was sonicated using an E220 focused ultrasonicator (Covaris, LLC; Woburn, MA, USA) to produce 200 bp fragments, which were purified using Agencourt AMPure XP beads (Beckman Coulter, Inc.; Indianapolis, IN, USA). Ovation Ultralow System V2 (Tecan Group Ltd.; Männedorf, Switzerland) was used to prepare Illumina libraries following the manufacturer’s instructions. Libraries were quality-checked for size and concentration with electrophoresis using a high-sensitivity D1000 kit on a 4200 TapeStation (Agilent Technologies, Inc.; Santa Clara, CA, USA). Sequencing depth was at 60 million (2 × 150 bp) paired-end reads per sample.

Metagenome reconstruction and analysis were performed on the U.S. Department of Energy Systems Biology Knowledgebase (KBase) platform [57] following the published protocol [58] using default parameters unless otherwise specified. Briefly, after importing the data files to KBase, read quality was assessed using FastQC (v0.12.1), followed by data pre-processing steps: trimming Illumina adapters, trimming low-quality bases on both read ends, removing duplicate reads, and removing host sequences with JGI RQCFilter pipeline (BBTools v38.22); then filtering out low-complexity reads (entropy filtering method with 0 ≤ 70 ≤ 100 entropy threshold or 0 ≤ 7 ≤ 100 dust threshold parameters) with PRINSEQ (v0.20.4). Metagenome assembly was performed using MEGAHIT (v1.2.9 [59]) with meta-sensitive parameter preset and default contig length parameter in the 300 ≤ 2000 range. Resulting contigs were binned using three methods: MaxBin2 (v2.2.4 [60,61] using 107 bacterial marker gene set), MetaBAT2 (v1.7 [62]), and CONCOCT (v1.1 [63] using BBMap mapping tool). Binned contigs from these three methods were optimized using DAS Tool (v1.1.2 [64] using blast [65] identification tool) to obtain consensus metagenome-assembled genomes (MAGs), which were evaluated with CheckM (v1.0.18 [66]) using a full reference tree for placement of each genome bin to a lineage clade for phylogenetic marker determination. The resulting data were filtered with CheckM to obtain medium-quality bin subsets with completeness ≥ 50% and contamination < 10% to maximize our detection of bile acid metabolism-associated genes. Functional summaries for each MAG were annotated using DRAM (v0.1.2 [67]), and taxonomy was assigned with GTDB-Tk (v2.3.2 [68]) using The Genome Taxonomy Database (GTDB) [69].

### 2.9. Statistical Analysis

All statistical analyses were conducted using R (v4.4.3) [70], unless otherwise specified in aforementioned sections. Results of the total bile acid assays, qPCR, and Western blot were analyzed by *t*-test within the rstatix package [71]. Significance was accepted at the level of *p* < 0.05 and *q* < 0.05. Respective visualizations were built using ggpubr [72] and plotly [73] packages.

## 3. Results

### 3.1. Bean Consumption Elevates the Excretion of Total Bile Acid Levels

**Figure 1:**
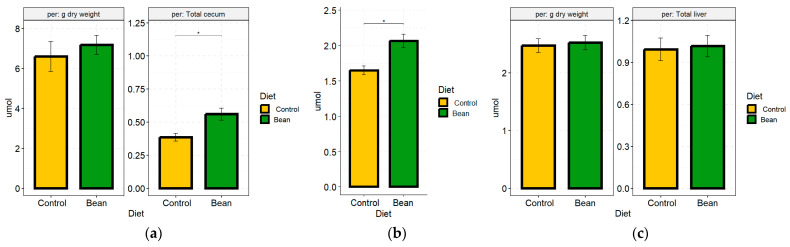
Bar plots of total bile acids in bean (green) and control (yellow) group in (**a**) cecal content, (**b**) feces, and (**c**) liver tissue samples. Left panel indicates amounts of total bile acids in μmol/g dry matter analyzed; right panel indicates μmol total bile acids in total tissue per animal (**a**,**c**). * indicates *p*-value < 0.05.

To determine the effects of cooked bean consumption on the levels of bile acids, cecal and fecal material, plasma, and liver tissue, biospecimens were subjected to an enzymatic total bile acid analysis from female mice fed a diet with ~60% of total dietary protein derived from bean. Plasma samples contained bile acids below the detection level for the assay, so we could not quantify their amounts. In cecal contents, there was no statistical difference between bean and control groups in the amount of total bile acids per g of dry weight (Figure 1a). However, we observed larger ceca owing to a greater amount of cecal contents in the bean group compared to the control groups across our studies (Supplementary Figure S1). Therefore, when we adjusted the values by the total cecal weights (bile acid concentration × cecum weight), we observed a statistically significant increase in total bile acid content in the bean group (Figure 1a). Consistent with this observation, bean consumption increased the amount of excreted bile acids compared to the control per g feces of dry weight (Figure 1b). These data indicate that bile acids are sequestered in the gut of bean-fed mice, and that a significant fraction of them escape enterohepatic circulation and are eventually excreted. In liver tissues, despite a trend of greater levels of total bile acids in the bean group, no statistical difference was observed (Figure 1c), indicating that the liver maintains homeostasis of bile acid content with no evidence of cholestasis.

### 3.2. Global Metabolomics Indicate a Higher Variety of Bile Acids upon Bean Consumption

**Figure 2:**
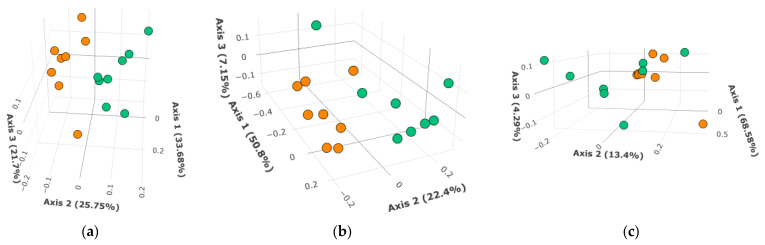
PCoA plots of bile acids in bean (green) and control (orange) samples using Bray–Curtis dissimilarity in (**a**) liver, (**b**) cecal, and (**c**) plasma samples. The 2D plots were adjusted to maximize visual separation of bean-fed and control samples.

Cecal content, liver tissue, and plasma samples were subjected to untargeted metabolomics analysis to gain deeper insight into bile acid changes that occur upon bean consumption. The work shown below was performed in female and male mice fed the same dietary formulations as in the previous section: the experimental group contained ~60% of total dietary protein derived from cooked common bean. The first question addressed was whether differences in relative abundance of identified bile acids by diet group (control or bean) were sufficient for separation in principal coordinates analysis (PCoA) using Bray–Curtis dissimilarity ordination (Figure 2) and Jaccard distances (Supplementary Figure S3).

Separation of liver samples was confirmed by PERMANOVA (Bray–Curtis: *R*^2^ = 0.243, *F* = 4.501, *p*-value = 0.001; Jaccard: *R*^2^ = 0.225, *F* = 4.071, *p*-value = 0.001). Similarly, differences were observed in the cecal metabolites (Bray–Curtis: *R*^2^ = 0.191, *F* = 3.306, *p*-value = 0.015; Jaccard: *R*^2^ = 0.196, *F* = 3.421, *p*-value = 0.003). Only plasma bile acids did not show a difference between control and bean (Bray–Curtis: *R*^2^ = 0.131, *F* = 2.108, *p*-value = 0.099; Jaccard: *R*^2^ = 0.125, *F* = 2.007, *p*-value = 0.082), which might be due to missing values in a lot of plasma samples (on average, 32%). Separation of bean and control samples was also statistically observed when primary and secondary bile acids were analyzed separately in the liver and cecum, but not plasma (Supplementary Figures S3 and S4). No differences according to PERMDISP tests were detected in the analyzed datasets, indicating that observed differences were not due to dispersion of data.

Collectively, these data signify marked differences in bile acid composition induced by bean consumption. Secondary bile acids may be of greater importance in driving these differences, judging by higher values of *F*-statistics compared with primary bile acid analysis in PERMANOVA. Global plasma metabolomics reflected our total bile acid assay results in plasma samples from a different study, indicating that bean effects on bile acids are more prominent within the gastrointestinal tract and liver rather than systemically.

**Figure 3:**
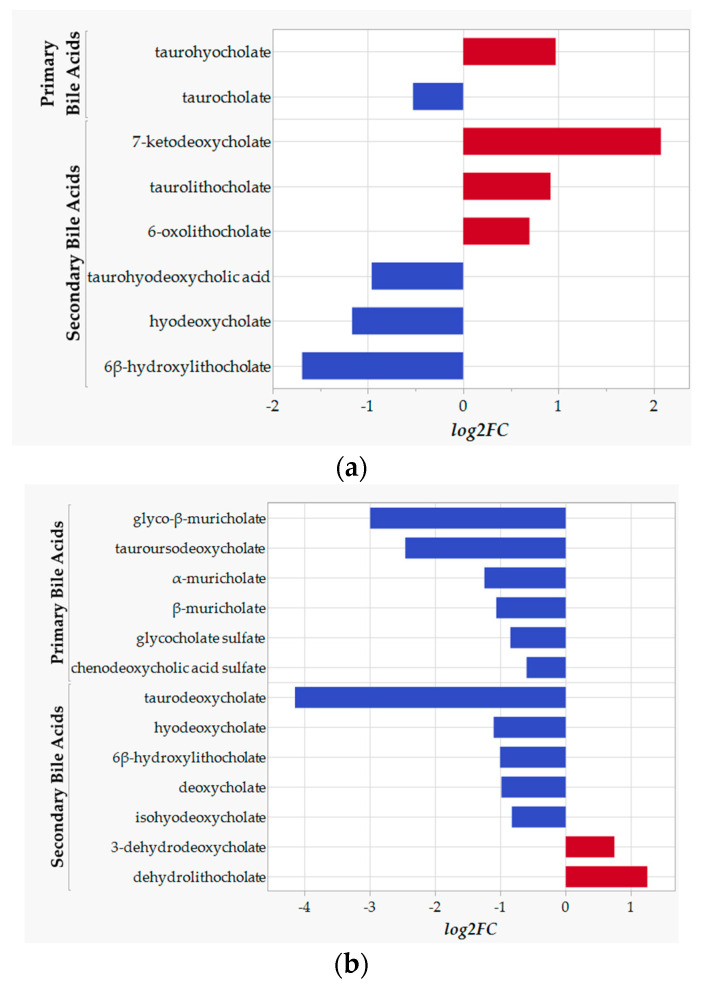
Bar plots of bile acid changes in bean versus control samples in (**a**) liver and (**b**) cecal samples. Only metabolites with *q* < 0.1 are depicted. Blue color indicates a decrease and red color indicates an increase in bean compared to control.

Next, we calculated the statistical differences in bean versus control groups to elucidate particular bile acids driving the differences between the control- and bean-fed animals (Figure 3; Supplementary Table S2). While metabolomics data of the liver tissue provide only a snapshot in time of bile acids produced and enterohepatically recirculated, the data indicate that among 15 detected primary bile acids, levels of taurocholate were reduced, while taurohyocholate was increased in the bean-fed animals (Figure 3a). With 0.1 > *p*-value > 0.05, glyco-β-muricholate tended to decrease, while taurochenodeoxycholic acid (7 or 27)-sulfate—to increase in livers of bean-fed animals (Supplementary Table S2). Conversely, 10 secondary bile acids were identified: animals that were fed bean exhibited lower levels of 6β-hydroxylithocholate, hyodeoxycholate, and taurohyodeoxycholic acid, whereas taurolithocholate, 6-oxolithocholate, and 7-ketodeoxycholate were significantly increased compared to bean-free control in the liver tissue.

In the cecal contents (Figure 3b), out of 15 detected primary bile acids, 6 were significantly decreased compared to the bean-free control, namely tauroursodeoxycholate, α- and β-muricholates, glyco-β-muricholate, glycocholate sulfate, and chenodeoxycholic acid sulfate. Interestingly, glycine conjugation of bile acids is atypical for rodents, pointing to the potential effect of gut microbial metabolism and respective reconjugation of bile acids under bean consumption [20,22]. Eighteen secondary bile acids (i.e., microbial metabolites) were identified in the metabolomics analysis of the cecal contents. Bean consumption significantly decreased levels of hyocholate derivatives: hyodeoxycholate and isohyodeoxycholate. Moreover, dehydrolithocholate and 3-dehydrodeoxycholate were elevated, indicating higher activity of bacteria harboring 3α-hydroxysteroid dehydrogenase activity metabolizing cholic acid. 6β-hydroxylithocholate, also known as murideoxycholate, was significantly reduced in the bean group, inferring either higher microbial 7α-dehydroxylation of β-muricholate, 6β-epimerization of hyodeoxycholate, or 6β-hydroxylation of lithocholate activities in control-fed animals [18,74,75]. Its reduction was also observed in the liver (Figure 3a). The reduction in taurodeoxycholate was the greatest in effect size, albeit it did not reach a significant *q*-value (Supplementary Table S2). With *p =* 0.078, 3-dehydrocholate showed a trend for an increase in bean-fed ceca.

Fewer differences were observed in plasma bile acids (Supplementary Table S2 and Figure S5). Presumably, this is due to the fact that not all samples had detectable signals for bile acids, which contributed to a lack of significant clustering and separation of the data seen in the PCoA (Figure 2c). Among 12 identified primary and 10 secondary bile acids, only taurochenodeoxycholate and 7-ketodeoxycholate approached a significant increase in the bean-fed animals, both approaching *p =* 0.05 cut-off but with insignificant *q*-values. However, taurochenodeoxycholate was detected only in half of the control samples. Secondary taurodeoxycholate and ursocholate also showed a trend to increase, while taurohyodeoxycholic acid—to decrease, but with 0.1 > *p*-value > 0.05.

These data demonstrate that bean consumption significantly alters the qualitative composition of bile acids in the cecal contents, liver, and plasma. The statistical emphasis appears to be skewed towards secondary bile acids as more differences in their levels were observed in mice that consumed bean.

### 3.3. Bean Affects Host Synthesis of Bile Acids

**Figure 4:**
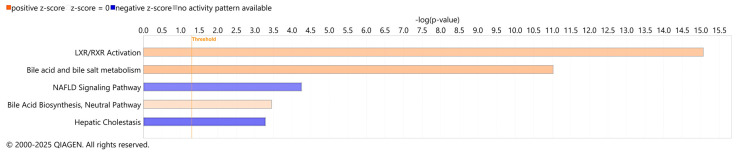
Canonical pathways analysis of bean-induced gene expression patterns associated with bile acid metabolism. Colors represent activation (shades of orange) or inhibition (shades of blue) of respective pathway. Features were deemed statistically significant with both activation |*z*|-score > 2 and *p*-value of overlap <0.05.

RNA-seq analysis of liver tissue samples from males and females of the same study that untargeted metabolomics were performed on was queried for effects on bile acid synthesis. DEGs in the RNA-seq data overlapped with a number of canonical pathways associated with the bile acids (Figure 4). Patterns of gene expression affected by consumption of bean in liver tissue indicated increased bile acid and bile salt metabolism (*z*-score = 3.962, *p*-value = 9.45 × 10^−12^) and bile acid biosynthesis (*z*-score = 2, *p*-value = 3.56 × 10^−4^). Liver X receptor and retinoid X receptor (LXR/RXR) activation also reached marked upregulation by bean with *z*-score = 3.55 and *p*-value = 8.57 × 10^−16^, which is consistent with LXR function inducing bile acid synthesis [20] and with observed higher levels of its agonist taurohyocholate in the liver [76,77]. Concomitantly, the pathway for hepatic cholestasis—a disorder associated with the accumulation of bile acids in the liver—was downregulated based on the gene expression data in the bean group (*z*-score = −2.466, *p*-value = 5.18 × 10^−4^). Similarly, the MASLD signaling pathway was also inhibited based on the patterns of bean-induced hepatic gene expression (*z*-score = −2.16, *p*-value = 5.53 × 10^−4^).

Canonically, bile acid synthesis is regulated by FGF15-FXR-SHP signaling. Synthesis of FGF15 is induced by bile acids in the small intestine as a feedback loop to activate the FXR-SHP pathway in the liver and suppress the synthesis of bile acids. In RNA-seq analysis, we detected a slight increase in *Nr1h4 (Fxr)*, *Nr0b2 (Shp)*, and FGF15 receptor *Fgfr4* levels, albeit with minor effect size (Table 1).

**Figure 5:**
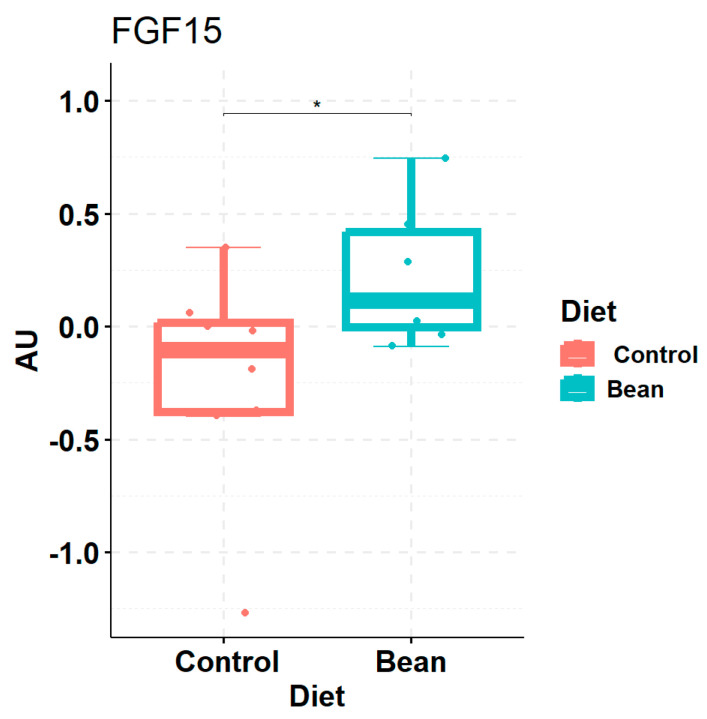
Box plot of protein levels of FGF15 from liver samples of animals fed control and bean diets. * indicates significant *p* < 0.05. AU—arbitrary units of signal peak area.

When we analyzed the protein level of FGF15 in the liver, we observed a minor increase (log_2_FC = 0.567) in bean vs. control (Figure 5). Although the antibodies for FXR and SHP in the liver were of insufficient quality, qPCR analysis of hepatic *Nr1h4 (Fxr)* and *Nr0b2 (Shp)* did not achieve significant *p*-values to confirm their increase in the liver (Table 2).

**Figure 6:**
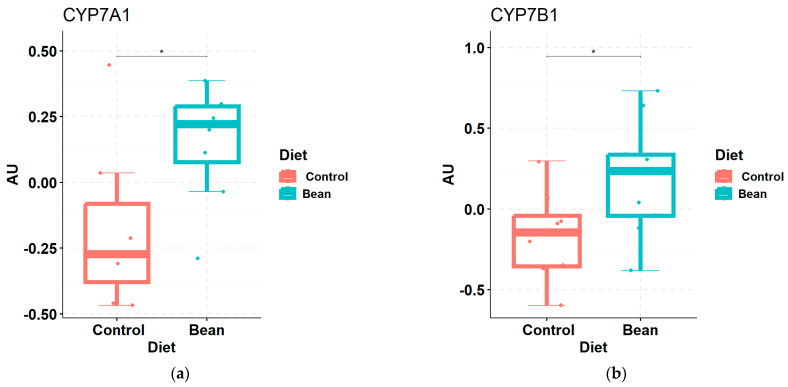
Box plots of protein levels of rate-limiting enzymes in bile acid biosynthesis: (**a**) CYP7A1 and (**b**) CYP7B1 in the liver samples of bean vs. control. * indicates significant *p* < 0.05. AU—arbitrary units of signal peak area.

FGF15-FXR-SHP signaling regulates bile acid synthesis upstream, so we looked into genes directly involved in bile acid production. In mice, 75% of primary bile acids are synthesized in the classical pathway, the rate-limiting enzyme of which is CYP7A1. Analogically, CYP7B1 catalyzes the rate-limiting step in the acidic pathway of 25% of primary bile acids in mice. Both enzymes perform 7α-hydroxylation reaction, albeit on different targets: CYP7A1 acts directly on cholesterol, while CYP7B1—on oxysterols [78]. RNA-seq analysis of the liver tissue in the bean-fed mice revealed that expression of both *Cyp7a1* (log_2_FC = 1.06, *p*-value = 1.1 × 10^−11^, *q*-value = 1.09 × 10^−9^) and *Cyp7b1* (log_2_FC = 1.14, *p*-value = 2.23 × 10^−15^, *q*-value = 5.39 × 10^−13^) is markedly enhanced compared to the control. This increase was also confirmed on the protein level (Figure 6).

**Figure 7:**
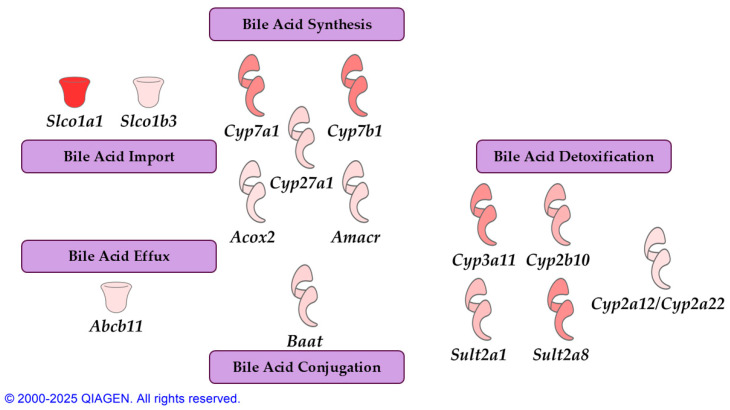
RNA-seq results on DEGs associated with bile acid metabolism in the liver tissue samples of bean-fed mice. Shades of red indicate log_2_FC. All depicted DEGs had *q* < 0.05.

Moderate upregulation was observed in downstream genes within bile acid biosynthetic pathways, such as *Cyp27a1, Amacr,* and *Acox2* (Figure 7; Supplementary Table S3). To reduce the hydrophobicity of bile acids and retain them in the lumen of the small intestine, bile acids are conjugated to taurine in mice by hepatic *Baat*, whose expression was also slightly increased by bean. Expression of *Cyp3a11* (ortholog of human CYP3A4), which produces hyocholic acid from chenodeoxycholic acid [20] and can bypass *Cyp7b1* and *Cyp27a1* in the acidic pathway [79], was also markedly enhanced by bean. RNA-seq analysis of liver tissue has also revealed that *Slco1a1* and *Slco1b3* (coding organic anion transporters OATP1 and OATP1B3, respectively) and to a smaller extent *Abcb11* (BSEP) were expressed more in bean-fed animals, suggesting higher capacity to import free bile acids into the liver and to efflux bile salts out of the hepatocytes, respectively. Unlike humans, mice rely more on phase I hydroxylation for detoxification of bile acids, where *Cyp3a11* takes an important part [21] together with *Cyp2b10*, which was also upregulated in mice. Nevertheless, mouse-specific *Cyp2a12/Cyp2a22,* which also enables rehydroxylation of secondary bile acids back to primary ones, was increased by bean in the liver [20,80], indicating that livers of the bean-fed animals might deal with an increase in secondary bile acids. However, sulfonation (phase II), albeit a minor detoxification pathway in mice [21], may be enhanced in bean-fed mice owing to higher expression of *Sult2a1* and *Sult2a8* (Supplementary Table S3).

### 3.4. Bean Consumption Enhances Microbial Contribution to the Bile Acid Metabolism

Most bile acids affected by bean consumption appear to be secondary and thus derive from microbial metabolism. Bacteria are capable of deconjugating hydrophilic bile salt into the hydrophobic form of bile acid, releasing the amino acid moiety. Using functional predictions from 16S rRNA gene data, we observed a slight increase in the primary and secondary bile acid biosynthesis pathway (ko00120, ko00121; log_2_FC = 0.15, *p*-value = 2.09 × 10^−4^, *q*-value = 5.05 × 10^−4^). Bile salt hydrolase (i.e., choloyglycine hydrolase, K01442, EC:3.5.1.24) upon bean consumption was also increased (Table 3), indicating a higher potential of bean-induced microbiome to deconjugate bile salts. Within the *bai* operon responsible for bacterial transformation of primary bile acids into secondary, functional predictions revealed only a significant reduction in NADH:flavin-dependent oxidoreductase (Table 3), i.e., NAD^+^-dependent 7β-hydroxy-3-oxo bile acid-CoA-ester 4-dehydrogenase (*baiH*). This enzyme catalyzes the intermediate step in dehydroxylation of ursodeoxycholic acid (the 7β-epimer of chenodeoxycholic acid) metabolites and other 7β-hydroxy bile acids [81]. Such a marked reduction in predicted *baiH*-carrying bacteria infers that bean consumption promotes dehydroxylation of bile acids rather at the 7α position, instead. Bean consumption also predicted a small reduction in 7α-hydroxysteroid dehydrogenase (7α-HSDH, *hdhA*, K00076, EC:1.1.1.159), which is involved in 7-O-epimerization of deconjugated cholate and chenodeoxycholate into ursocholate and ursodeoxycholate, respectively. This is consistent with the observed reduction in taurine-conjugated ursodeoxycholate in the ceca of the bean-fed animals. PICRUSt2 also predicted increased bile acid:Na^+^ symporter in the cecal microbiome of bean-fed animals (Table 3), consistent with our observations that bean-induced bacteria have a higher capacity to import and metabolize bile acids.

**Figure 8:**
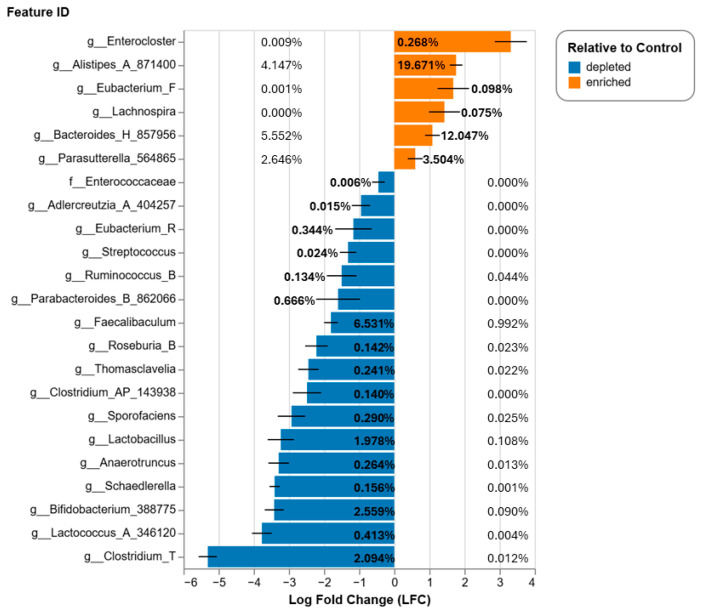
Results of differential abundance analysis of cecal microbiota in bean vs. control using ANCOM-BC. Only genera associated with bile acid metabolism and *q*-value < 0.05 are displayed. The numbers on each bar denote average relative abundance of bacterium in control (on the left) and in bean (on the right) groups. Additional details on ANCOM-BC can be found in Supplementary Table S4.

Among the putative bacterial genera metabolizing bile acids [24,82,83,84], we observed an overall reduction in more taxa than increased upon bean consumption (Figure 8), consistent with our previous report [85]. However, relative abundances of bean-enhanced bacteria were higher than those suppressed, particularly *Alistipes, Bacteroides*, and *Parasutterella*. On average, the cumulative abundance of bile acid-associated bacteria was 28.442% in control and 37.183% in bean, indicating higher microbial capacity, albeit lower diversity, to metabolize bile acids. This is particularly important considering that bean-fed animals also exhibited larger ceca with more cecal content (Supplementary Figure S1); thus, the net output of microbial contribution to bile acid metabolism in the colon of mice and their exposure to microbial metabolites of bile acids are even greater.

**Figure 9:**
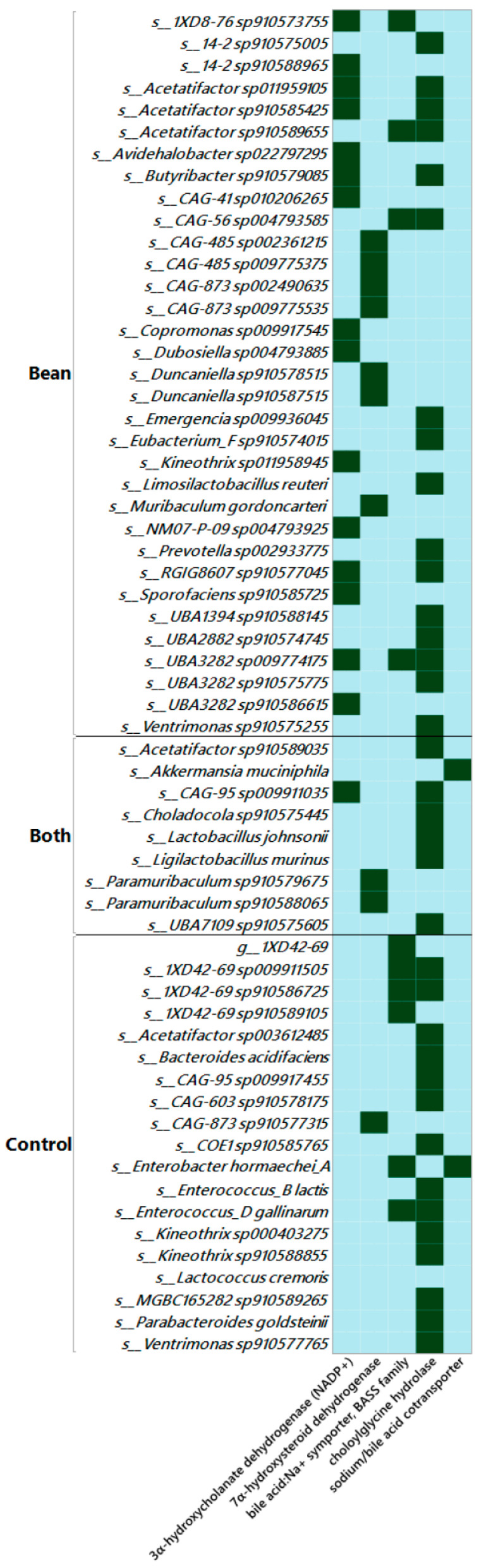
Summary of bile acid-associated genes found in the shotgun metagenomics analysis. Green color indicates the presence of respective enzyme in identified MAGs. Choloylglycine hydrolase [EC:3.5.1.24; K01442]; 3α-hydroxycholanate dehydrogenase (NADP^+^) [EC:1.1.1.392; K22605]; 7α-hydroxysteroid dehydrogenase [EC:1.1.1.159; K00076]; bile acid:Na^+^ symporter, BASS family [K03453]; sodium/bile acid cotransporter [SLC10A7; K14347].

In a different study, we applied shotgun metagenomic analysis on the cecal contents of bean and control diet-fed mice. Using liberal quality controls, we identified 61 MAGs in the control and 73 MAGs in the bean group (Supplementary Table S5). After mapping to the Genome Taxonomy Database (GTDB), we identified 19 unique taxa in control, 33 in bean, and 9 shared by both (Figure 9). Some of these taxa were not canonically recognized as bile acid-metabolizing bacteria, and the presence of bile salt hydrolase was the largest (36 hits out of 74), perhaps indicating horizontal transfer of the gene encoding this enzyme, which has been reported [86,87]. Additionally, only bean MAGs possessed NADP^+^-dependent 3α-hydroxycholanate dehydrogenase (EC:1.1.1.392, K22605).

## 4. Discussion

The role of bile acids in modulating many aspects of metabolic dysfunction associated with the obesity pandemic is receiving considerable attention [18,78]. Because the metabolic pathways regulated by bile acids, e.g., cholesterol metabolism and lipid homeostasis, overlap with the reported effects of consumption of therapeutic amounts of cooked bean [88,89], the proof-of-concept experiments reported herein were conceived. We expected that the consumption of cooked bean would bind bile acids based on several reports that bean components sequester bile acids as detected using in vitro assays [90,91,92] and in vivo [93,94,95]. The data in Figure 1 are consistent with the hypothesis that cooked bean sequesters bile acids, resulting in increased fecal bile acid elimination, which is considered a definitive biomarker for bile acid sequestration [26,96]. Dietary fiber, protein, and even bean phytosterols are capable of binding bile acids [27,97,98]. This differs from earlier reports, indicating that only the soluble component of dietary fiber sequesters bile acids [99,100] and is of particular importance in view of the fact that about 60% of bean fiber is insoluble [29]. Moreover, bean and other pulses have an amount of protein in essentially a 1:1 ratio (*w*/*w*) with dietary fiber [11]. Thus, the fact that bean protein also sequesters bile acids is significant [27]. Bean protein has a lower digestibility than familiar animal sources of the protein, suggesting that protein-sequestered bile acids arrive in the colon, where a mixture of fiber and protein undergoes microbial metabolism, rendering bile acids accessible for microbial metabolism as well. The biotransformational chemistry is supported by enriched microbial ecogroups, which we have previously reported [85], and is therefore of interest relative to host exposure to gut microbiota-derived bioactive compounds. In this regard, the effects of the bean are expected to be different than clinically prescribed bile acid sequestering resins, either of the first (cholestyramine and colestipol) or second generation (colesevelam) that precipitate bile acids in the intestine, and those complexes are nonreactive and are excreted [26,96].

While bean consumption increased bile acid excretion in the feces, it also increased the total bile acid content in the cecum, the first segment of the colon (Figure 1). Principal coordinates analysis (PCoA) of the bile acid data from global metabolomic analyses showed complete separation between the bean and the control group (Figure 2; Supplementary Figures S2–S4). Further examination of these data indicated that conjugated primary bile acids were deconjugated in the cecum as well as converted to secondary bile acids. Thus, at least some of the bile acids reaching the colon are biologically active and participate in biotransformation reactions within the cecal microbiome.

Nonconjugated bile acid metabolites can be absorbed via passive diffusion in the colon and transported in the blood to the liver and other tissues, where they have been reported to exert hormone-like effects. Of particular interest in this regard are recently published data showing that caloric restriction of mice in the same mouse model resulted in increased conversion of primary bile acids to derivatives of lithocholic acid [101]. Reported changes in mTOR pathway signaling were partly mediated by the induction of AMP-activated protein kinase (AMPK) by lithocholate, a secondary bile acid. This observation has particular significance in that we have reported that bean feeding limits weight gain in obesogenic rodent models, an effect also observed in response to caloric restriction [32,39]. The effects of bean consumption on weight regulation have also been reported in population studies [102,103]. Moreover, we have previously reported the activation of AMPK by bean consumption in rat models of diet-induced obesity and breast cancer [17]. In addition, the AMPK-regulated node of the mTOR signaling network, which is also induced by metformin, is associated with longevity extension [101,104]. Bean consumption has also been associated with longevity extension in human population centers called blue zones [105].

Studies in preclinical models to identify differences in the relative abundance of microbial taxa of bean-fed versus control mice generally target fecal material [106,107,108]. Our work has moved the focus of analysis from the stool, which reflects the end stage of microbial impacts on the host, to the cecal content, recognizing that the cecum is the first segment of the colon at the end stage of host digestion. In a series of studies in rodents, a consistent relandscaping of the cecal microbiome has been observed in response to bean consumption, and variation across studies in the induced, unaffected, and suppressed bacterial genera and species have also been noted, a common observation in microbiome research [108]. However, herein, we reported 16S rRNA gene amplicon data (Figure 8) and shotgun metagenomics (Figure 9) that provide evidence of an enhanced ability to metabolize bile acids in bean-fed mice. Moreover, the metagenomic data provide insights for future follow-up experiments, i.e., the primers designed to permit quantitative assessment of bean-driven effects at the species and strain taxonomic level using methods like those proposed in [109].

Given these observations, we predicted that the liver’s FXR and SHP transcript levels would not differ or be reduced in bean-fed versus control mice. qPCR data showed no significant difference (Table 2). Next, we found strong evidence for an increased rate of bile acid synthesis in bean-fed mice via both the canonical (produces both cholic and chenodeoxycholic acid) and the acidic pathway (produces chenodeoxycholic acid). We assessed protein levels of CYP7A1 and CYP7B1 hydroxylases, rate-limiting steps in bile acid synthesis, the synthesis of which is inhibited by FGF15-FXR-SHIP signaling [110,111]. This finding is consistent with our previous work, which showed that transcript levels of *Cyp7a1* were also induced by feeding a similar amount of cooked bean in a rat model [112]. The combined data indicate that this effect is observed in two rodent species and is consistent with the expectation that bile acid sequestration by bean would not be species-dependent. Chenodeoxycholic acid serves as a precursor for other primary bile acids, such as hyocholic and ursodeoxycholic. These differences were consistent with the global metabolomic analyses data, which showed complete separation between bile acid profiles of bean-fed versus control-fed mice (Figure 2). However, it is important to note that total bile acids were measured in the liver, and no differences were observed between the bean- and control-treated mice (Figure 1c). This finding indicates bean does not induce bile acid accumulation in the liver due to cholestasis, a condition where the flow of bile from the liver to the small intestine is impaired, which can also result in higher circulating levels of bile acids. This is consistent with observed RNA-seq patterns of increased transport of bile acids to (OATP1/3) and from (BSEP) hepatocytes (Supplementary Table S3). These findings are also consistent with our previous report that no evidence of hepatotoxicity was observed in rats fed cooked bean in the diet [112]. The caveat is that the total bile acid assay relies on the 3α-hydroxysteroid dehydrogenase enzymatic reaction and thus would not detect epimers with 3β-OH and other 3α-dehydroxylated metabolites of bile acids. This underscores the importance of targeted bile acid analyses that detect a broad range of metabolites, given the diversity of bile acids recently reported to be generated during microbial metabolism [24,113,114]. Bean-induced microbial biotransformation of bile acids is not limited to just 7α-hydroxylation, as evidenced by the increase in dehydro-, keto-/oxo-forms of secondary bile acids. Moreover, bean upregulates the mouse host capacity to rehydroxylate secondary bile acids. We also showed a relative decrease in cholate and deoxycholate with a shift towards their microbial metabolites, potentially carrying a health benefit, given that an increase in these two 12α-hydroxylated bile acids has been associated with metabolic morbidities [78,115]. Finally, we show that bean consumption affects the metabolism of health-associated bile acid—hyocholic acid [77]—by increasing hepatic taurohyocholate yet reducing its microbial metabolites in the liver and cecum.

Therefore, bean must modulate bile acid metabolism beyond just sequestration and elimination of bile acids or altering microbial community and its net access to and capacity to metabolize bile acids. Previously, we have reported that feeding bean to mice and rats reduces serum levels of cholesterol and LDL [32], which was consistent with our later report that bean also impedes de novo synthesis of fatty acids and triglycerides and affects their packaging into lipid droplets and VLDL structures. Shown herein, the increase in bile acid synthetic pathways, their chelation, and elimination with fecal pellets suggest additional impact on cholesterol metabolism, redistributing its molecules towards bile acid metabolism and away from hepatic and potentially systemic lipotoxicity. Understandably, this would require future work on fasted animals to elucidate further bean effects on cholesterol synthesis, biotransformation, and circulating levels regulation. Moreover, additional multi-focal studies are required to outline which effects of bean consumption are direct (driven by dietary components of cooked bean and its host-mediated metabolites) versus indirect (mediated by the activity of bean-induced microbial ecogroups). The latter, one might say, would also include changes in host physiology owing to changes induced by direct effects of bean components and their metabolites. For example, here, we show direct effects of dietary bean components on sequestering bile acids and increasing the colonic bacteria exposure to them, which, in turn, would affect cholesterol metabolism and absorption of other lipids in the lower small intestine. Therefore, it is important to understand that the multifactorial effect of bean consumption should not be underestimated yet must be accounted for in the future studies of its mechanisms.

### Limitations

The work reported herein was retrospective, using biospecimens from preclinical studies designed for other purposes. While this is a strength in some respects, quantitative collection of feces for three to five days, which is recommended for assessing bile acid sequestration in rodent models, was not undertaken. In addition, it needs to be recognized that host exposure to various bile acid metabolites needs to be assessed. This will require a targeted analysis of a comprehensive panel of bile acid metabolites in which values are normalized to the dry matter weight of the biospecimen and further corrected for differences in mass of the luminal content or amount of feces excreted. For the cecum and presumably other colon segments, the luminal content mass generally exceeds that of the control by a factor of 1.5 or more in bean-fed rodents. Moreover, the same argument needs to be considered in quantifying the effects of bean on gut microbiota since relative abundance data limit insights concerning host exposure to specific microbiota and their products of metabolism [116]. Finally, to fully elucidate cardiometabolic health status, other derivatives of cholesterol, such as circulating lipoproteins (HDL, LDL, VLDL), need to be considered. Since our biospecimen collection was performed on animals not in a fasting state, any interpretation of cholesterol data was of limited value. However, future quantitative work on unraveling the effects of cooked bean consumption in an obesogenic environment on the bile acid composition and metabolism would benefit significantly from the data on fasted cholesterol levels not only in the serum, but also in the liver and gastrointestinal tract, given that cholesterol is also synthesized in the lower ileum.

## 5. Conclusions

Collectively, the data reported here provide strong support for the prospective investigation of bile acid mediation of bean-driven health benefits. The consumption of cooked bean modulates all levels of bile acid metabolism: their synthesis rate, host modification, and gut microbial modification. Given the chelating properties of bean-derived chyme in the small intestine, bean consumption potentially also affects lipid metabolism and thus the host’s absorption of cholesterol and other lipid species. Given that in most of our studies, mice are fed obesogenic diets (including the bean-containing ones), such multifactorial protection of animals from an obesogenic challenge points to the importance of this type of food as a source of plant-based protein, dietary fiber, and other bioactive food components.

## Figures and Tables

**Table 1:** RNA-seq results of hepatic *Fxr*-*Shp* signaling in bean vs. control.

DEG	ID	Expr Log Ratio	Expr *p*-Value	Expr *q*-Value
*Fgfr4*	ENSMUSG00000005320	0.27	0.00103	0.01
*Shp (Nr0b2)*	ENSMUSG00000037583	0.4	0.00017	0.0026
*Fxr (Nr1h4)*	ENSMUSG00000047638	0.3	0.000736	0.00848

**Table 2:** qPCR results of hepatic *Fxr* and *Shp* in bean vs. control.

Bean vs. Control				
Gene	Avg ΔCt Control	Avg ΔCt Bean	−ΔΔCt	*p*-Value
*Fxr (Nr1h4)*	2.2485	2.1105	0.138	0.387
*Shp (Nr0b2)*	3.798	3.2855	0.5125	0.523

**Table 3:** PICRUSt2-predicted metagenome of cecal microbiota in bean vs. control.

Gene/Enzyme	log_2_FC	*p*-Values	*q*-Values	ID
BSH, choloylglycine hydrolase	0.57	5.84 × 10^−31^	2.39 × 10^−30^	K01442
*hdhA*; 7-alpha-hydroxysteroid dehydrogenase [EC:1.1.1.159]	−0.22	7.62 × 10^−13^	6.12 × 10^−2^	K00076
*baiH*; NAD+-dependent 7beta-hydroxy-3-oxo bile acid-CoA-ester 4-dehydrogenase	−2.45	8.19 × 10^−49^	1.73 × 10^−47^	K15873
TC.BASS; bile acid:Na^+^ symporter, BASS family	0.98	7.59 × 10^−32^	3.29 × 10^−31^	K03453
SLC10A7, P7; solute carrier family 10 (sodium/bile acid cotransporter), member 7	−0.21	0.574	0.608	K14347

## Data Availability

The original contributions presented in this study are included in the article/Supplementary Material. Further inquiries can be directed to the corresponding author.

## Supplementary Materials

The following supporting information can be downloaded at: https://www.mdpi.com/article/10.3390/nu17111827/s1, Figure S1: box plot depicting cecal weights in bean-fed animals versus control; Figure S2: PCoA plots of bile acids in bean and control samples using Jaccard index; Figure S3: PCoA plots of primary bile acids in bean and control samples using Bray–Curtis dissimilarity and Jaccard distances; Figure S4: PCoA plots of secondary bile acids in bean and control samples using Bray–Curtis dissimilarity and Jaccard distances; Figure S5: box plots of identified bile acid metabolites in the liver tissue, cecal contents, and plasma samples of control- and bean-fed mice; Table S1: dietary formulations across studies; Table S2: statistical differences in the bile acid metabolites in bean vs. control; Table S3: DEGs associated with bile acid metabolism from RNA-seq analysis in bean vs. control; Table S4: ANCOM-BC results in bean vs. control with bile acid-metabolizing bacteria; Table S5: DRAM results in bean vs. control with bile acid-metabolizing genes.
