# Supplementary material for: Cooked Bean (Phaseolus vulgaris L.) Consumption Alters Bile Acid Metabolism in a Mouse Model of Diet-Induced Metabolic Dysfunction: Proof-of-Concept Investigation
Source: Nutrients. 2025 May 28;17(11):1827. doi: 10.3390/nu17111827 (PMC12157300; doi:10.3390/nu17111827)
Supplement: Supplementary file 1 [file nutrients-17-01827-s001.zip › Supplementary Figures Final.pdf]

Supplementary Figures

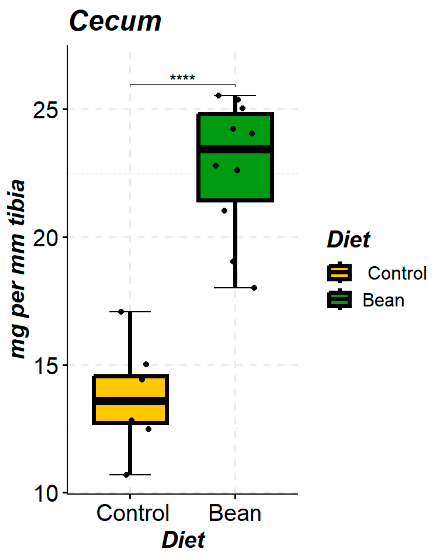

Figure S1. Box plot depicting cecal weights in bean-fed animals (green) versus control (yellow). **** indicates p-value < 0.0001. Cecal weights in mg were normalized to the tibia length in mm.

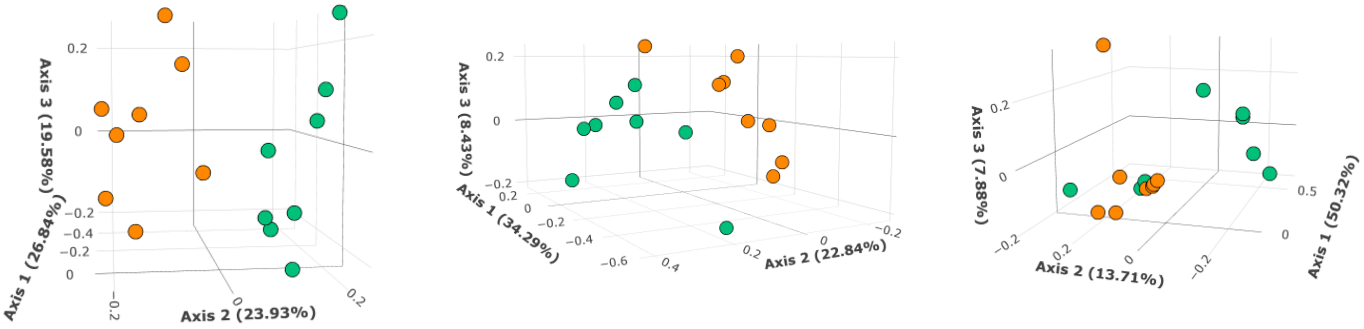

(**a**) (**b**) (**c**)

Figure S2. PCoA plots of bile acids in Bean (green) and Control (orange) samples using Jaccard index in (**a**) liver, (**b**) cecal, and (**c**) plasma samples.

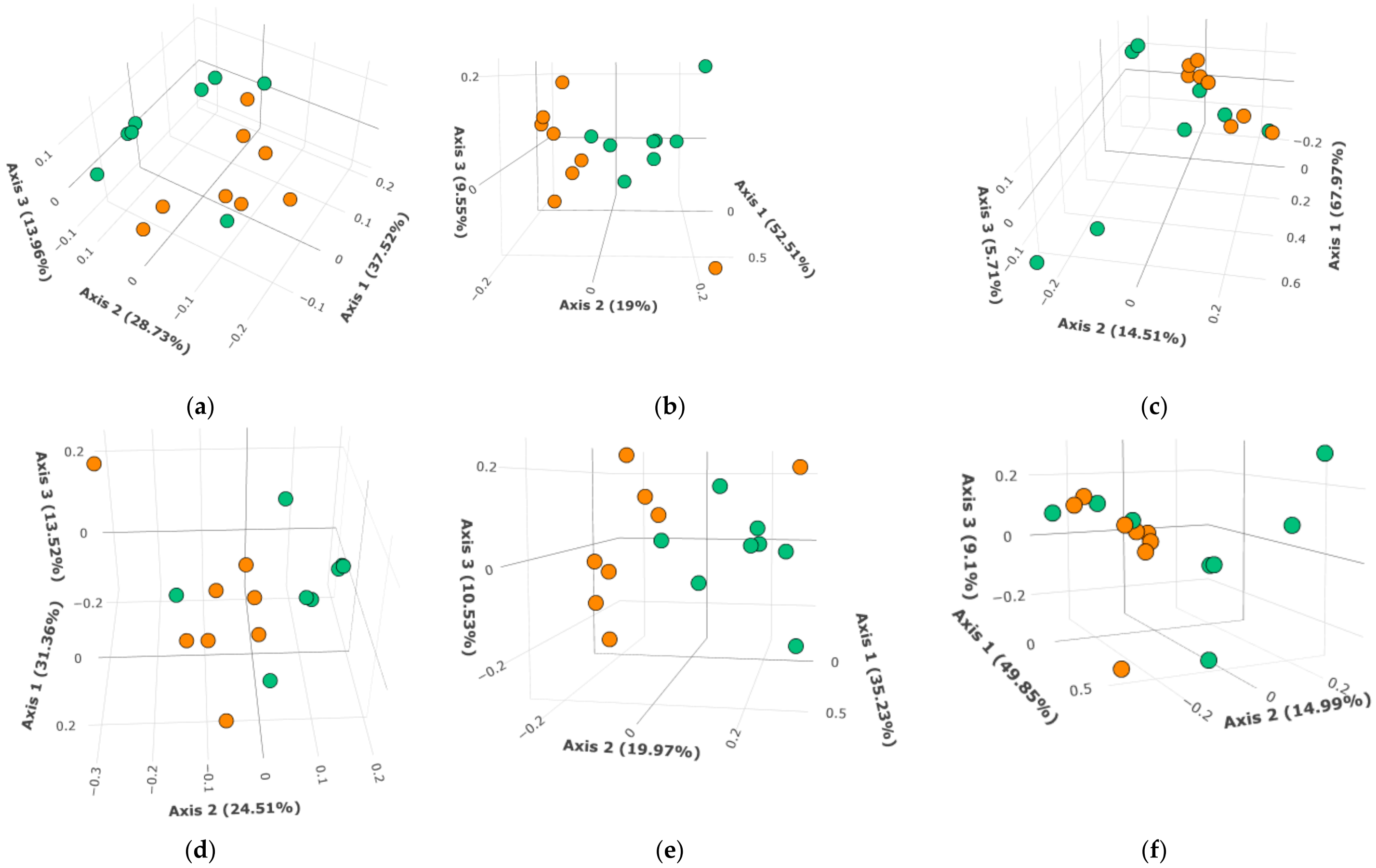

Figure S3. PCoA plots of primary bile acids in Bean (green) and Control (orange) samples using Bray-Curtis dissimilarity (**a**–**c**) and Jaccard (**d**–**f**) distances in (**a, d**) liver, (**b, e**) cecal, and (**c, f**) plasma samples.

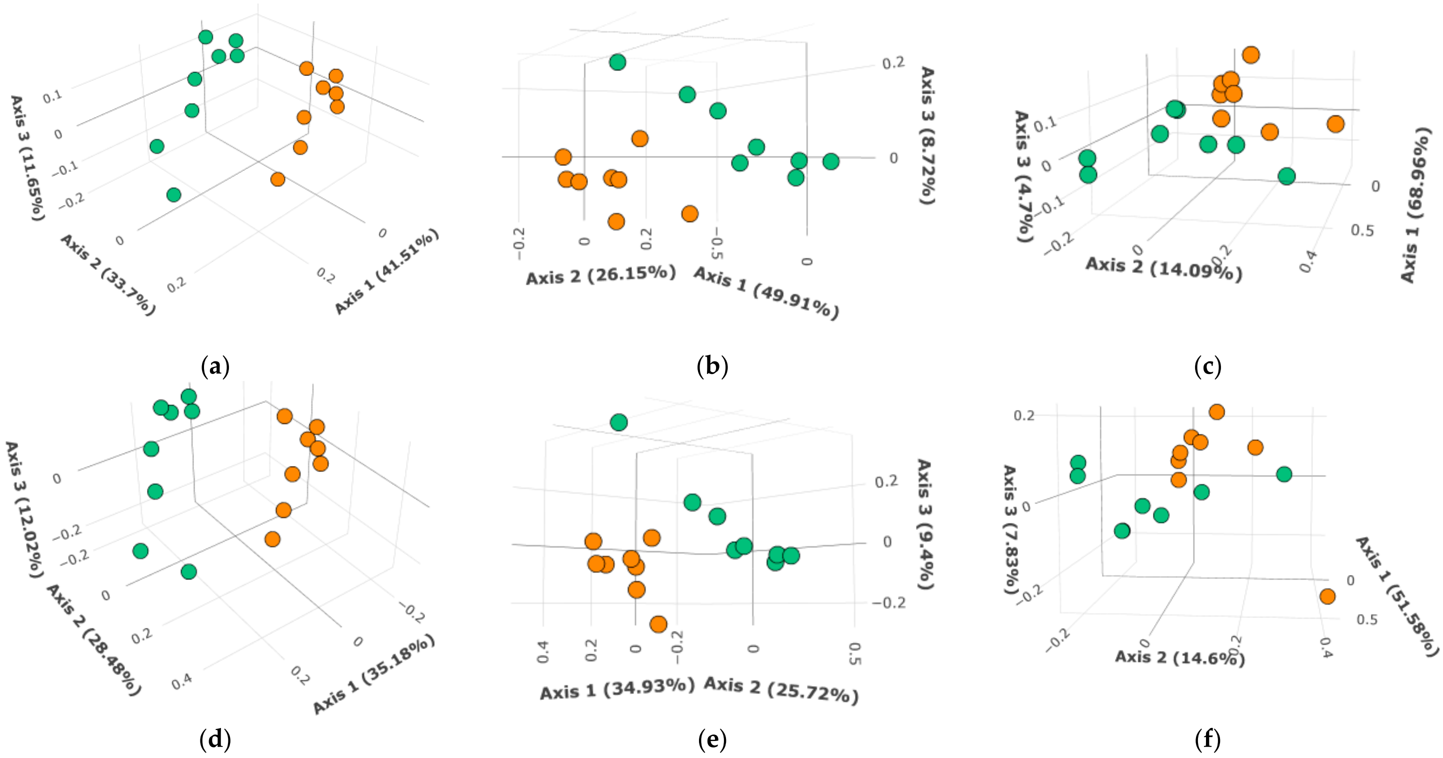

Figure S4. PCoA plots of secondary bile acids in Bean (green) and Control (orange) samples using Bray-Curtis dissimilarity (**a**–**c**) and Jaccard (**d**–**f**) distances in (**a, d**) liver, (**b, e**) cecal, and (**c, f**) plasma samples.

LIVER

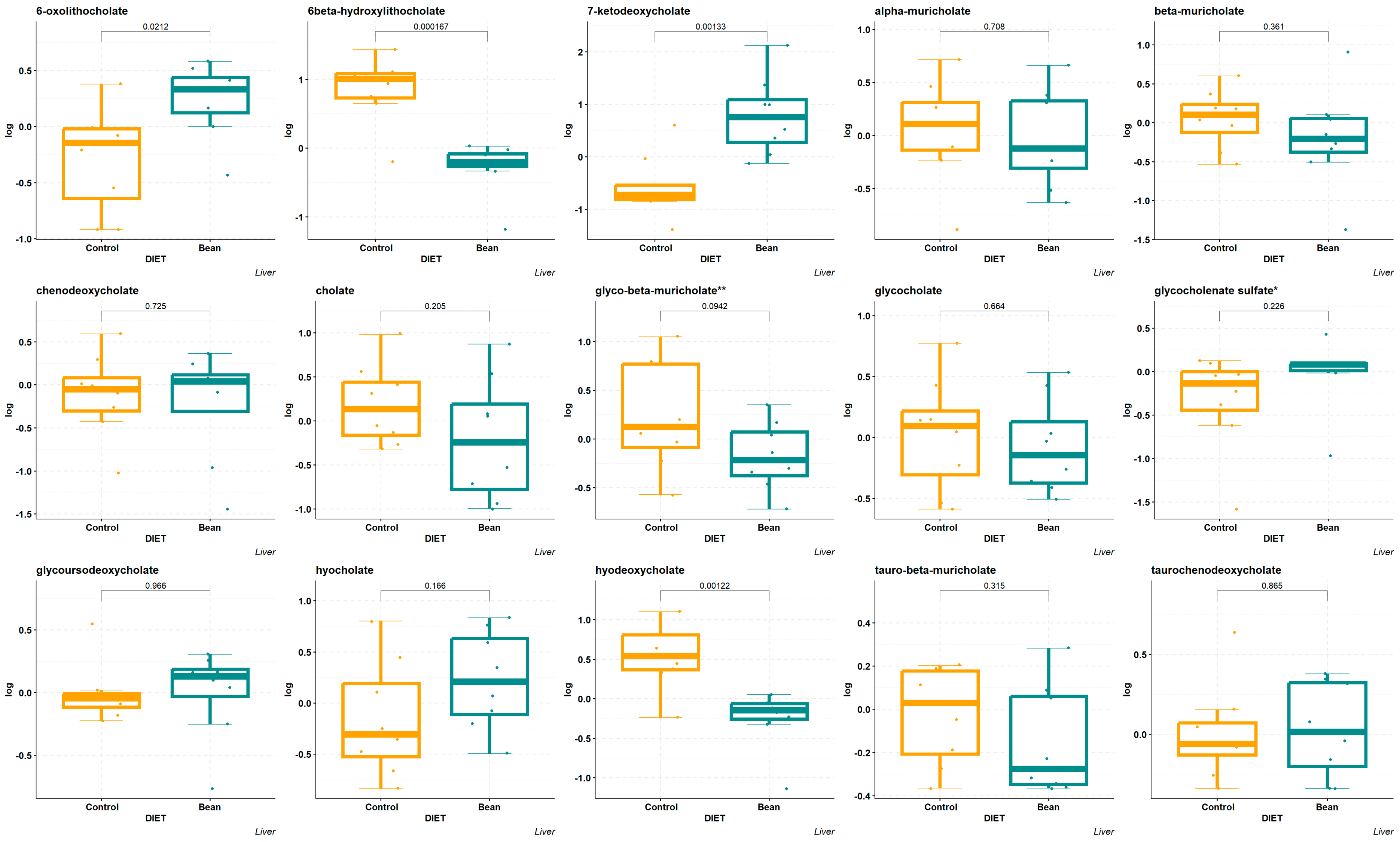
6-oxolithocholate
0.0212
6beta-hydroxylithocholate
0.000167
7-ketodeoxycholate
0.00133
alpha-muricholate
0.708
beta-muricholate
0.361
chenodeoxycholate
0.725
cholate
0.205
glyco-beta-muricholate**
0.0942
glycocholate
0.664
glycocholenate sulfate*
0.226
glycoursodeoxycholate
0.966
hyocholate
0.166
hyodeoxycholate
0.00122
tauro-beta-muricholate
0.315
taurochenodeoxycholate
0.865
log
Control
Bean
DIET
Liver

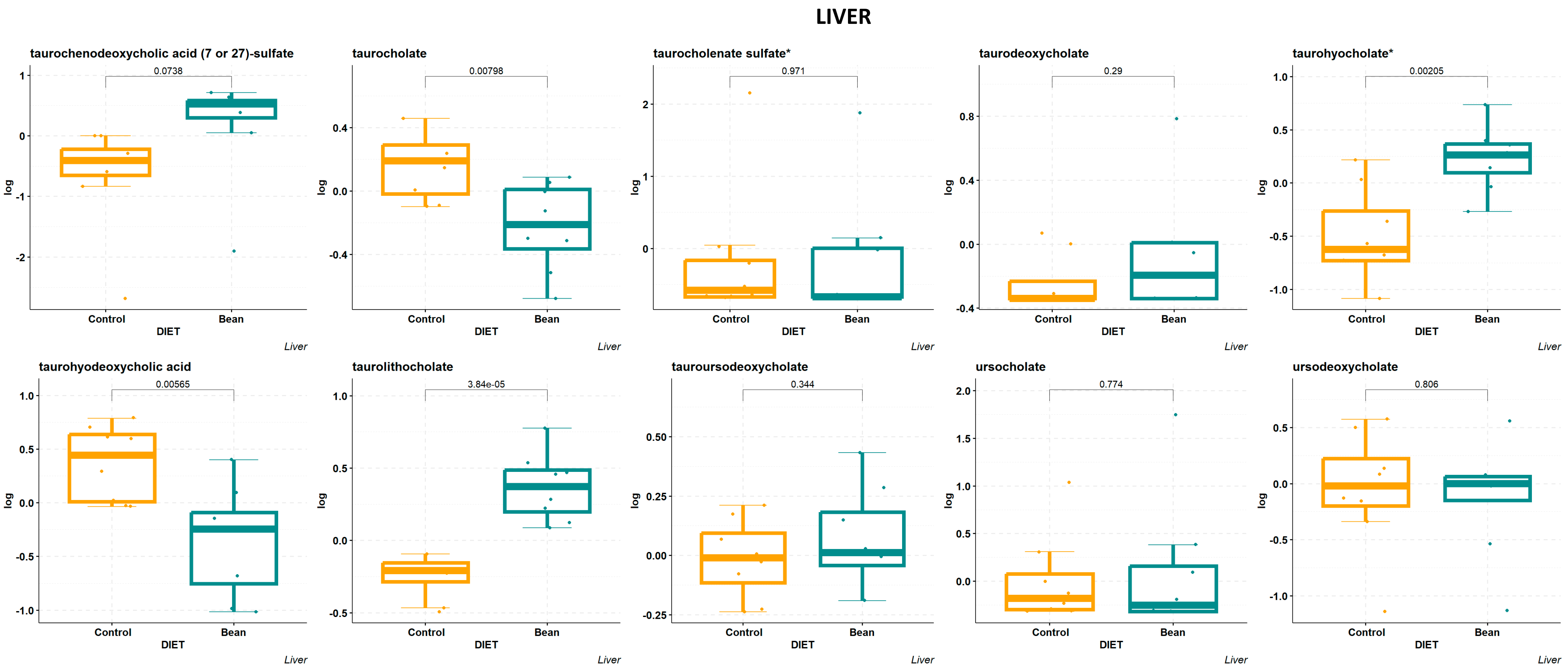
LIVER
taurochenodeoxycholic acid (7 or 27)-sulfate
0.0738
taurocholate
0.00798
taurocholenate sulfate*
0.971
taurodeoxycholate
0.29
taurohyocholate*
0.00205
taurohyodeoxycholic acid
0.00565
taurolithocholate
3.84e-05
tauroursodeoxycholate
0.344
ursocholate
0.774
ursodeoxycholate
0.806
log
Control
Bean
DIET
Liver

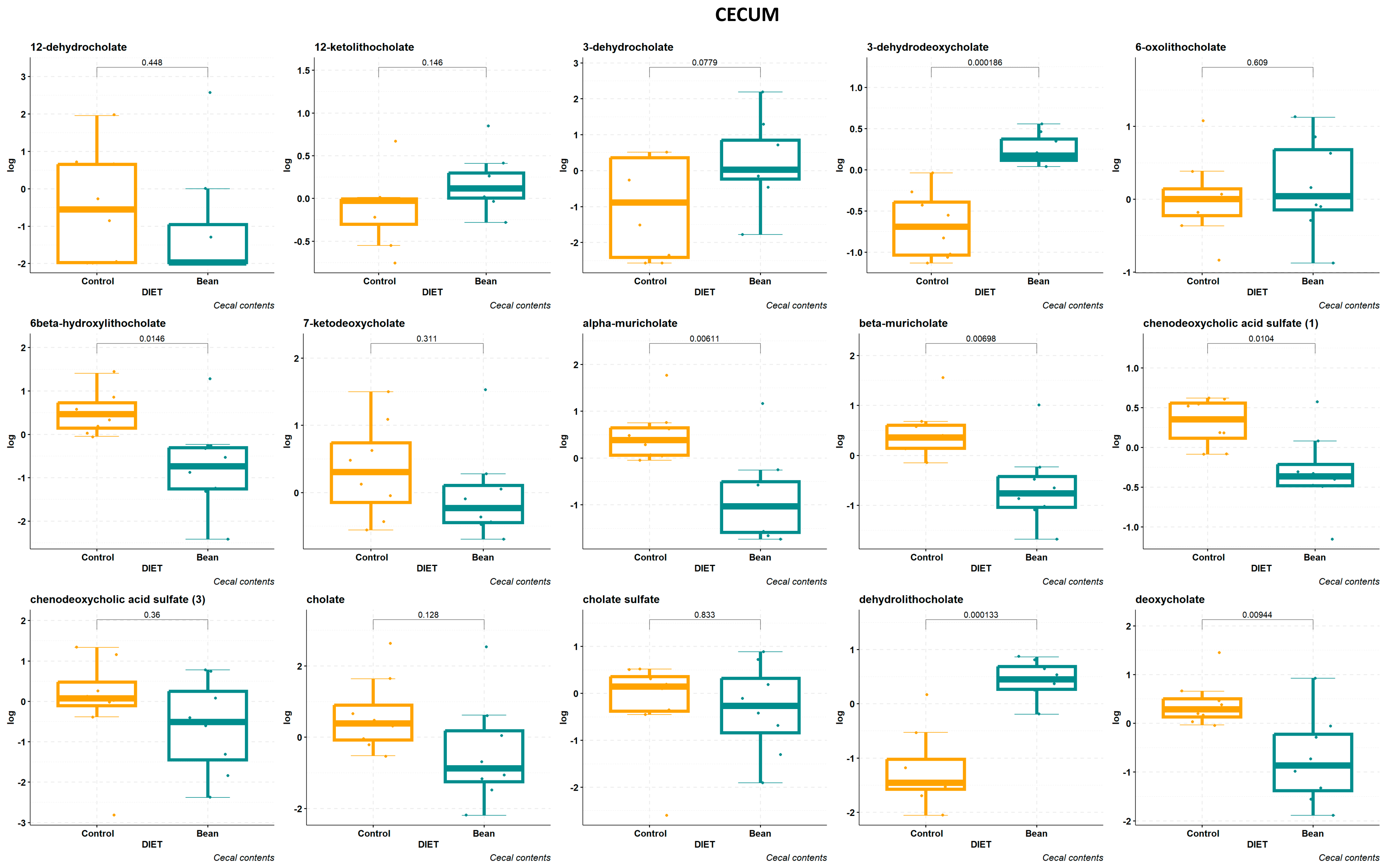
CECUM
12-dehydrocholate
0.448
12-ketolithocholate
0.146
3-dehydrocholate
0.0779
3-dehydrodeoxycholate
0.000186
6-oxolithocholate
0.609
6beta-hydroxylithocholate
0.0146
7-ketodeoxycholate
0.311
alpha-muricholate
0.00611
beta-muricholate
0.00698
chenodeoxycholic acid sulfate (1)
0.0104
chenodeoxycholic acid sulfate (3)
0.36
cholate
0.128
cholate sulfate
0.833
dehydrolithocholate
0.000133
deoxycholate
0.00944
log
Control
Bean
DIET
Cecal contents

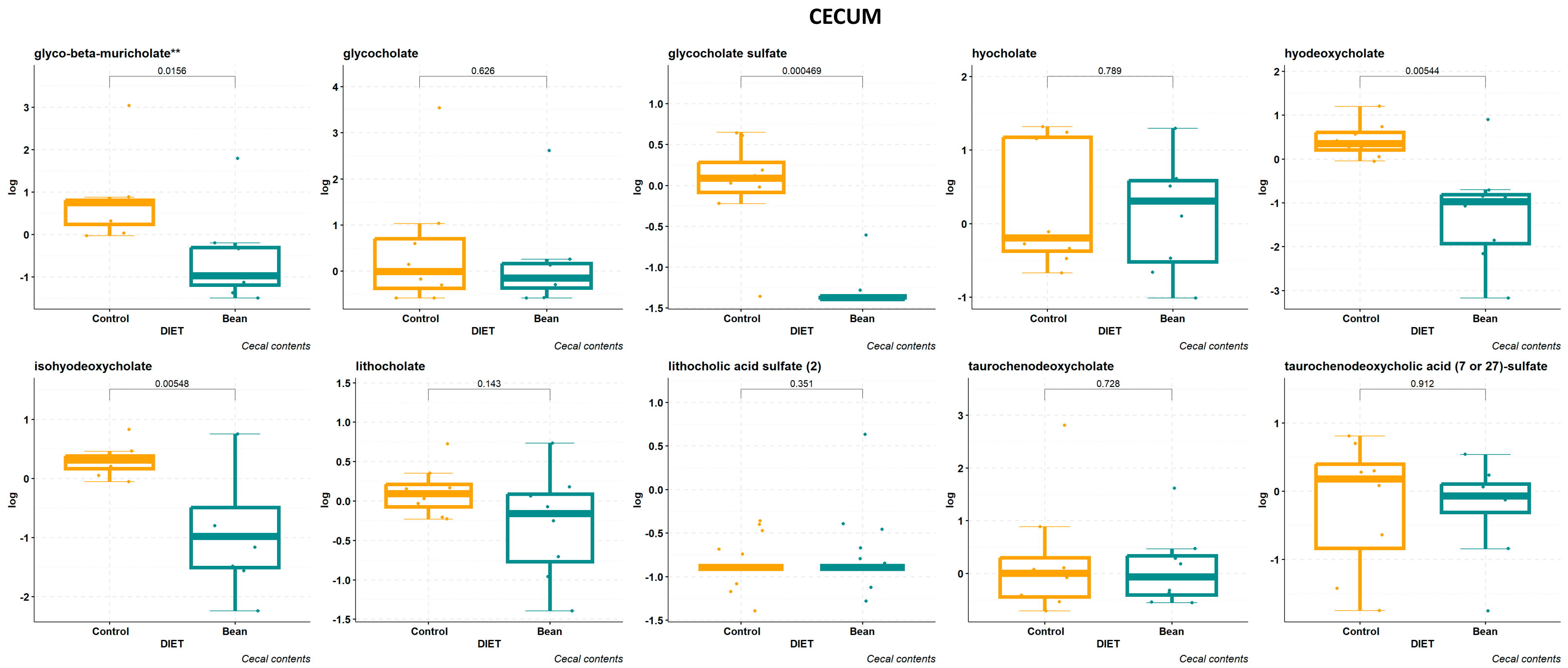
CECUM
glyco-beta-muricholate**
0.0156
glycocholate
0.626
glycocholate sulfate
0.000469
hyocholate
0.789
hyodeoxycholate
0.00544
isohyodeoxycholate
0.00548
lithocholate
0.143
lithocholic acid sulfate (2)
0.351
taurochenodeoxycholate
0.728
taurochenodeoxycholic acid (7 or 27)-sulfate
0.912
log
Control
Bean
DIET
Cecal contents

CECUM

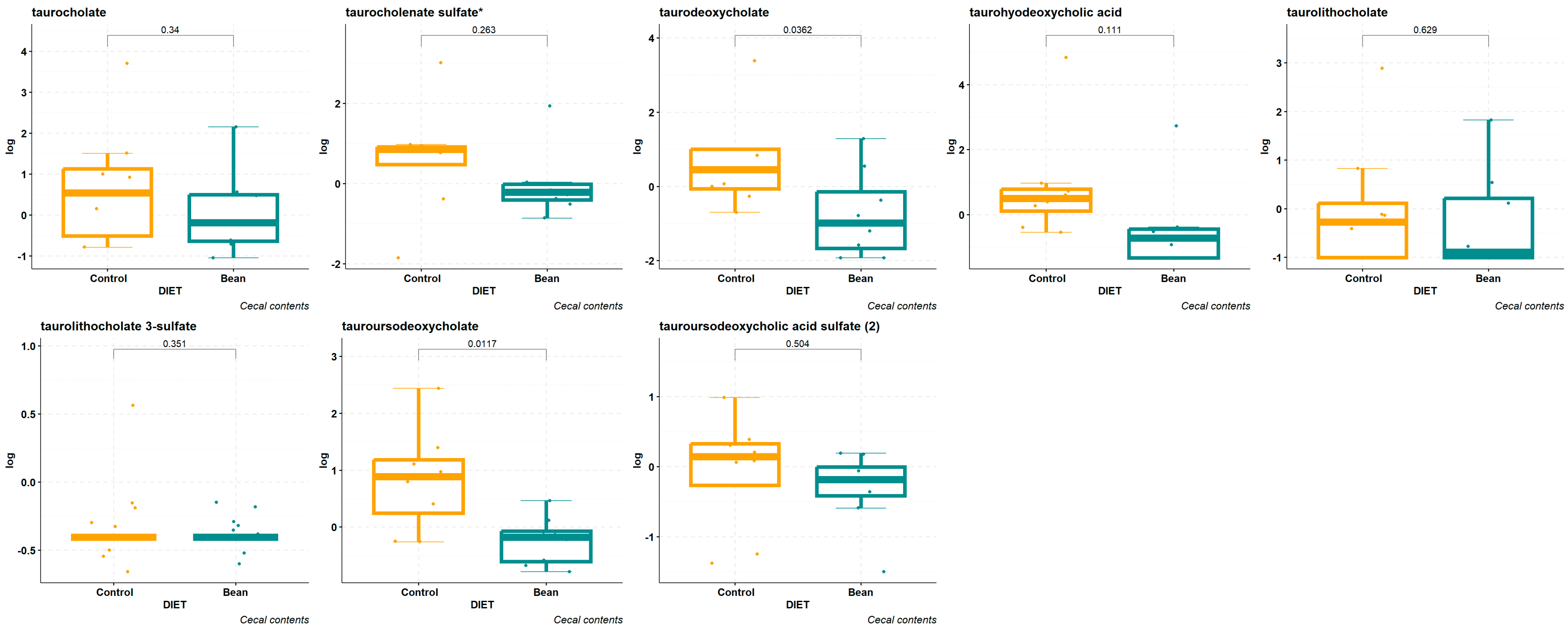

taurocholate
0.34
taurocholenate sulfate*
0.263
taurodeoxycholate
0.0362
taurohyodeoxycholic acid
0.111
taurolithocholate
0.629
taurolithocholate 3-sulfate
0.351
tauroursodeoxycholate
0.0117
tauroursodeoxycholic acid sulfate (2)
0.504
log
Control
Bean
DIET
Cecal contents

PLASMA

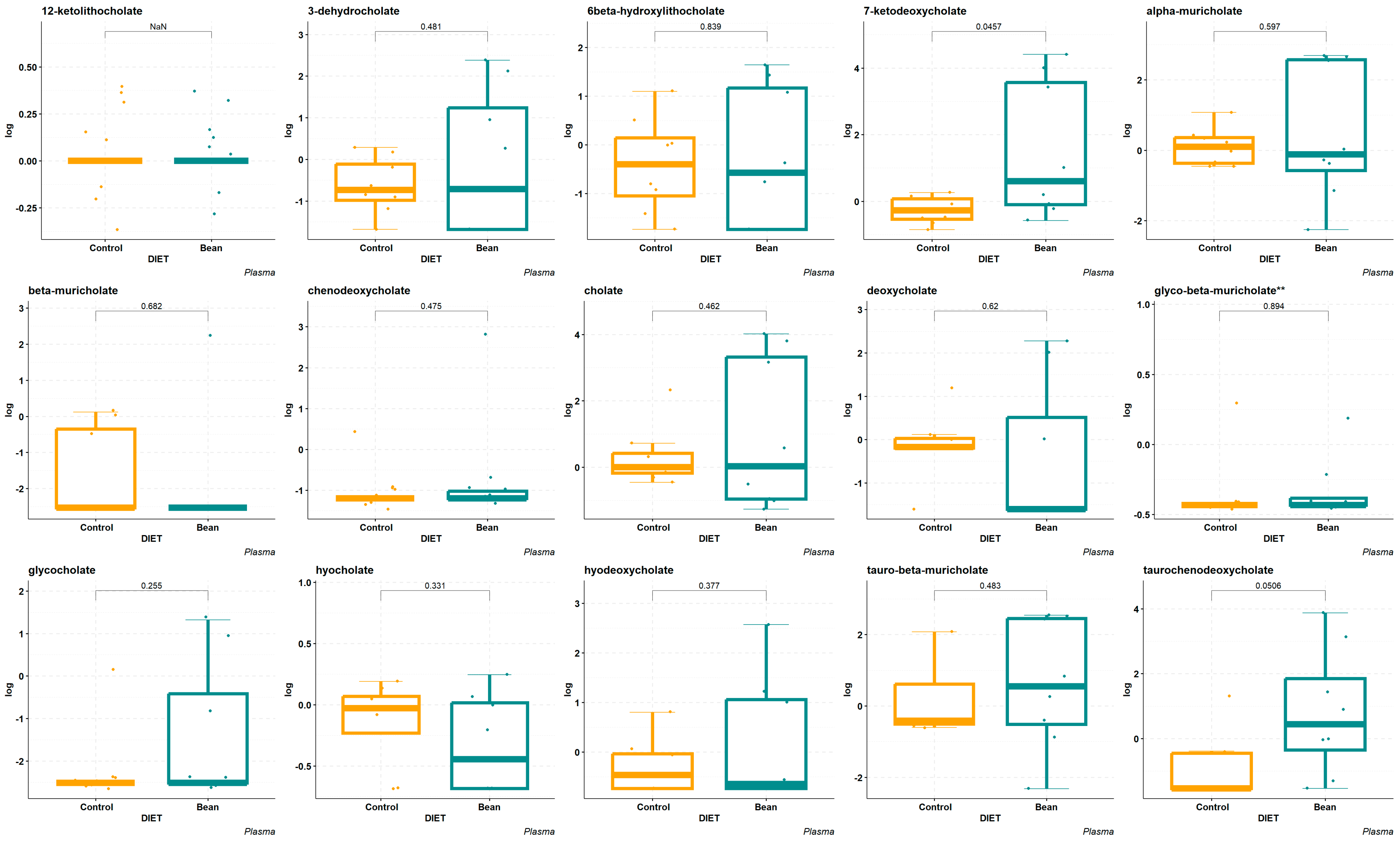

12-ketolithocholate
NaN
3-dehydrocholate
0.481
6beta-hydroxylithocholate
0.839
7-ketodeoxycholate
0.0457
alpha-muricholate
0.597
beta-muricholate
0.682
chenodeoxycholate
0.475
cholate
0.462
deoxycholate
0.62
glyco-beta-muricholate**
0.894
glycocholate
0.255
hyocholate
0.331
hyodeoxycholate
0.377
tauro-beta-muricholate
0.483
taurochenodeoxycholate
0.0506
log
Control
Bean
DIET
Plasma

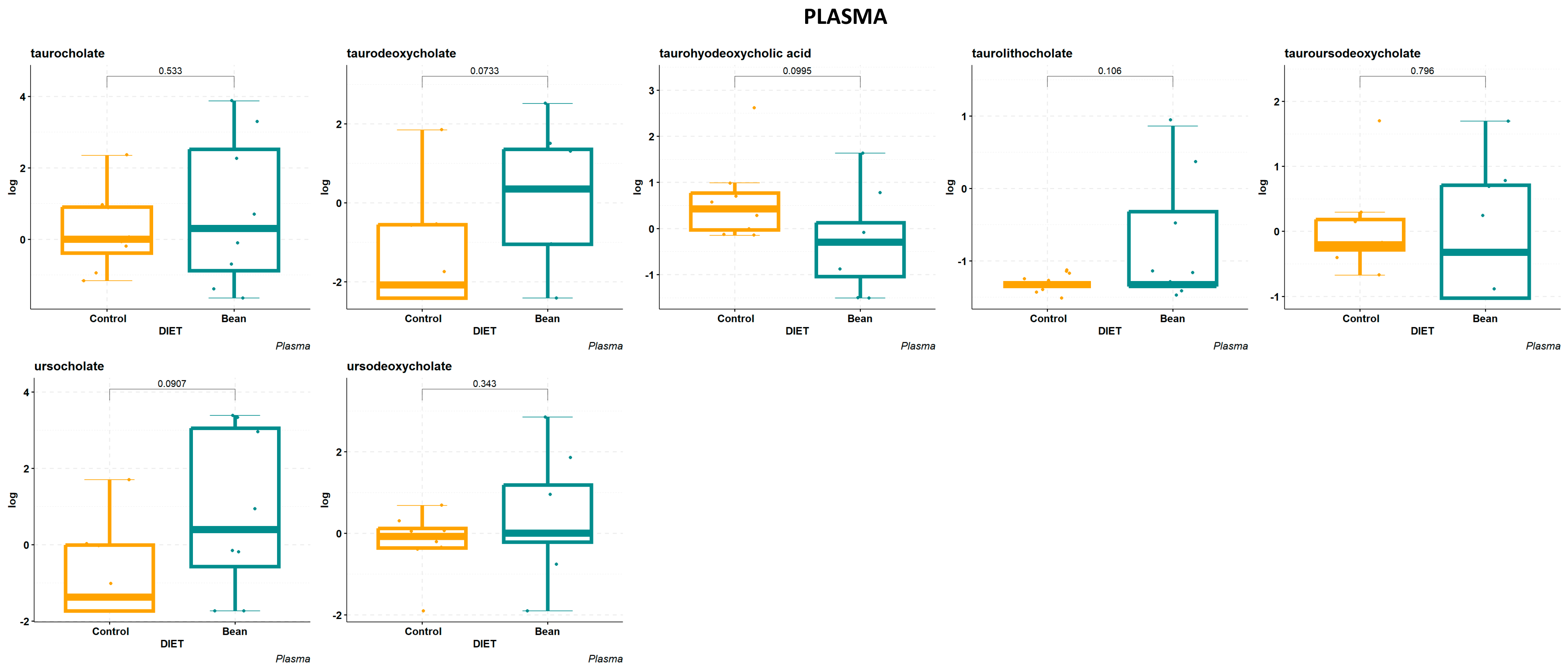

Figure S5. Box plots of identified bile acid metabolites in the liver tissue, cecal contents, and plasma samples of Control- and Bean-fed mice. Each tissue type is separated by section and indicated on the bottom-right of each plot. Metabolites *p*-values are indicated on top between each box pair.
